# Supplementary material for: Chromatogram libraries improve peptide detection and quantification by data independent acquisition mass spectrometry
Source: Nat Commun. 2018 Dec 3;9:5128. doi: 10.1038/s41467-018-07454-w (PMC6277451; doi:10.1038/s41467-018-07454-w)
Supplement: Supplementary file 1 — Supplementary Information [file 41467_2018_7454_MOESM1_ESM.pdf]

Supplementary Information for:

Chromatogram libraries improve peptide detection and quantification by data independent acquisition mass spectrometry.

Brian C. Searle[1,2], Lindsay K. Pino[1], Jarrett D. Egertson[1], Ying S. Ting[1], Robert T. Lawrence[1], Brendan X. MacLean[1], Judit Villén[1], and Michael J. MacCoss[1]*

[1]Department of Genome Sciences, University of Washington, Seattle, WA, USA
[2]Proteome Software, Portland, OR, USA
*Corresponding author, email: maccoss@uw.edu

SUPPLEMENTARY NOTE 1

PECAN(1), or PEptide-Centric ANalysis, is an algorithm for detecting peptides from DIA experiments directly without the use of spectrum libraries. While high-mass accuracy spectrum libraries for human samples and some model organisms are commonly available, sometimes spectrum libraries are either impossible to gather (with precious samples) or not cost-effective to generate for uncommon organisms. PECAN can be used to help ease that burden by removing the need for DDA-based spectrum libraries.

We have implemented a desktop-friendly version of the PECAN scoring system, named Walnut, to enable chromatogram library generation from FASTA protein sequence databases when spectrum libraries are unavailable. We have made minor modifications to the scoring algorithm that heavily optimize it for speed and memory consumption. These modifications result in different scores for some types of peptides compared to the original Python implementation but in general do not affect the performance over all. Walnut is packaged with EncyclopeDIA and the source code is available as part of the EncyclopeDIA repository on Bitbucket at: https://bitbucket.org/searleb/encyclopedia under the open source Apache 2 license.

SUPPLEMENTARY NOTE 2

We employed several steps generally following the "Large Scale DIA with Skyline" webinar (https://skyline.ms/webinar14.url) to ensure that Skyline (2) produced optimal results when analyzing DIA data. To perform DIA parameter optimization in Skyline, we used SkylineRunner and the batch scripts available here https://skyline.ms/webinars/Webinar14_scripts.zip. Based on preliminary analyses, we use 73 peptides as iRT standards that we consistently found across all the runs.

1. REPAEQPGDGER
2. RPLEDGDQPDAKK
3. QREEEIEAQEK
4. LQEKEELR
5. EGGDGEEQDVGDAGR
6. LSSETYSQAK
7. SDSGKPYYYNSQTK
8. TAEDSTAAMSSDSAAGSSAK
9. SYSSGGEDGYVR
10. LTVAENEAETK
11. LREDENAEPVGTTYQK
12. ETKPEPMEEDLPENKK
13. GPSAAGEQEPDKESGASVDEVAR
14. SGNYPSSLSNETDR
15. HITTISDETSEQVTR
16. GGGFGGNDNFGR
17. SADGSAPAGEGEGVTLQR
18. AFAAQEDLEK
19. LVKPGNQNTQVTEAWNK
20. ELEIESQTEEQPTTK
21. VGDDVEFEVSSDRR
22. GLQAPAGEPTQEASGVAAAK
23. GQLC[+57.0]ELSC[+57.0]STDYR
24. VAVEEVDEEGKFVR
25. TKPYIQVDIGGGQTK
26. ALQRPSAAAPQAENGPAAAPAVAAPAATEAPK
27. SQPVSQPLTYESGPDEVR
28. ASWSSLSMDEK
29. SQVFSTAADGQTQVEIK
30. SHVEDGDIAGAPASSPEAPPAEQDPVQLK
31. EDC[+57.0]EQWWEDC[+57.0]R
32. EDLRLPEGDLGKEIEQK
33. VLQSALAAIR
34. VQVQDNEGC[+57.0]PVEALVK
35. SVPTSTVFYPSDGVATEK
36. LIEGLSHEVIVSAAC[+57.0]GR
37. SFDLLVK
38. MTPPIKDLLPR
39. NLFEDQNTLTSIC[+57.0]EK
40. AIELFSVGQGPAK
41. GVTIIGPATVGGIKPGC[+57.0]FK
42. EEPGSDSGTTAVVALIR
43. EC[+57.0]SIYLIGGSIPEEDAGK
44. SQLDIIIHSLK
45. QTLMWSATWPK
46. VLIALLAR
47. IYGLGSLALYEK
48. GEAGVPAEFSIWTR
49. VFVLDEADVMIATQGHQDQSIR
50. VDILDPALLR
51. NPPGFAFVEFEDPR
52. IIDPLPPIDHSEIDYPPFEK
53. LRPLYDIPYMFEAR
54. LNDGHFMPVLGFGTYAPPEVPR
55. KIPNPDFFEDLEPFR
56. FLSQPFQVAEVFTGHMGK
57. GAGTNEDALIEILTTR
58. TLAQLNPESSLFIIASK
59. GIISILDEEC[+57.0]LRPGEATDLTFLEK
60. SSEMNVLIPTEGGDFNEFPVPEQFK
61. DLLGTVWGGPANLEAIAK
62. SLNTDVLFGLLR
63. FMQDASDVMQLLLK
64. GLLPEELTPLILATQK
65. EHMGNVVEALIALTN
66. EC[+57.0]LPLIIFLR
67. VIEPQYFGLAYLFR
68. EKVETELQGVC[+57.0]DTVLGLLDSHLIK
69. NYLDWLTSIPWGK
70. EIFLSQPILLELEAPLK
71. VTGQHPEVPPAFWNNAFTLLSAVSLPR
72. NLATAYDNFVELVANLK
73. AVLAPLIALVYSVPR

For the final analysis, we followed these steps:

- Configure document with chromatogram library
    - Add all peptides from chromatogram library (99,370 with 242 “unmatched”)
    - Edit > Reintegrate > Advanced min 6 transitions (94,560 peptides)
- Configure document with spectral library
    - Add all peptides from spectral library (140,590 with 25,764 “unmatched”)
    - Edit > Reintegrate > Advanced min 6 transitions (130,101 peptides)
    - Accept peptides with iRT values (128,741 remaining)
- Remove precursors and MS1 filtering from both documents (-nop)
- Reduce peptides to only the set contained in both libraries (91,024 remaining) (-reduced)
    - Accept peptides in both documents of list from the other – 3536 in clib not in blib
- Use chromatogram library iRTs with DDA library (-cirt)
- Use DDA iRTs with chromatogram library (-birt)

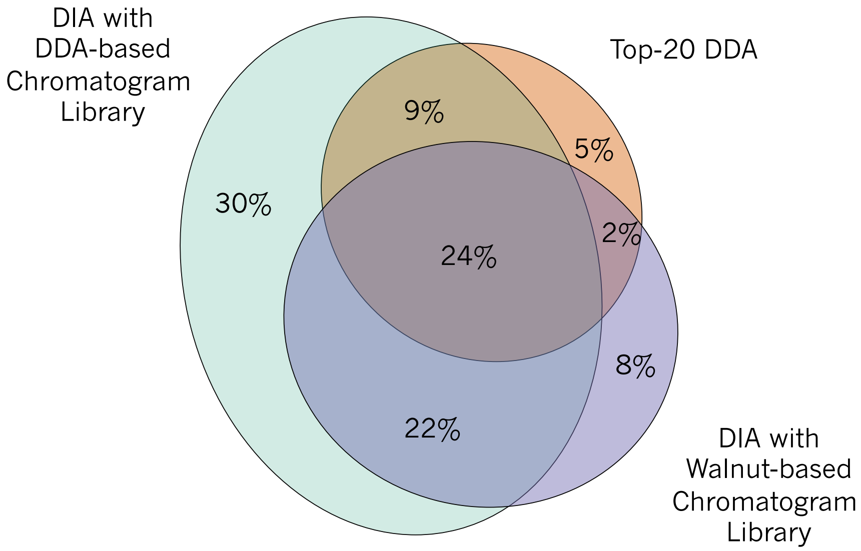

Supplementary Figure 1: Peptide detection overlap between DIA and DDA. An Euler diagram showing the overlap between unique peptide detections filtered at 1% FDR from a single HeLa replicate of either top-20 DDA or wide-window DIA searched with the DDA-based chromatogram library or the Walnut-based chromatogram library. EncyclopeDIA using a DDA-based chromatogram library detects 85% of peptides found using all three methods combined.

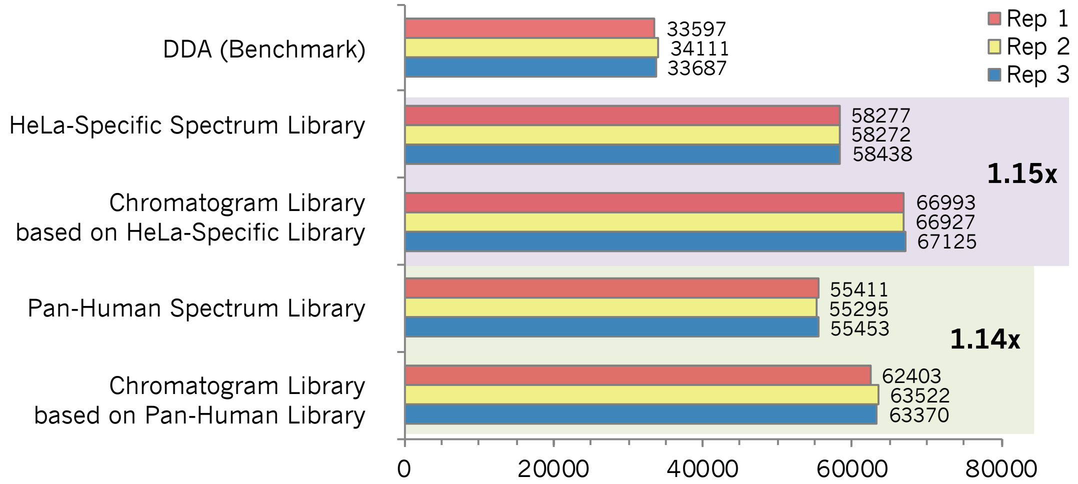

Supplementary Figure 2: Chromatogram libraries improve peptide detection rates using Skyline. The number of peptide detections at 1% peptide FDR in triplicate HeLa injections improve by approximately 1.15x with Skyline when using chromatogram libraries as compared to searching either the HeLa-specific or Pan-Human DDA-based spectrum libraries. Peptide detection results from top-20 DDA (searched with Comet) are used as a benchmark. Skyline library searches were performed using a 10 minute retention time extraction window and validated to a 1% peptide FDR with mProphet and applied without any run-to-run alignment.

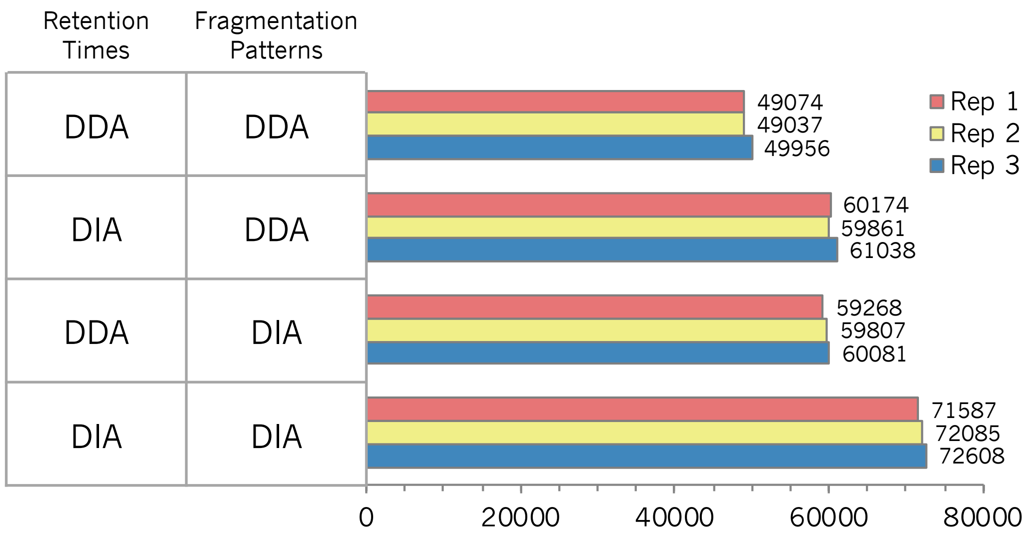

Supplementary Figure 3: Additive effect of retention time and fragmentation patterns. The number of peptides detected at 1% peptide FDR in triplicate HeLa injections using the HeLa-specific chromatogram library (DIA), where either the retention times or fragmentation patterns have been switched with the spectrum library (DDA). Compared to the original HeLa-specific spectrum library search (average of 47863 peptides), a small improvement (3%) comes from simply using a narrowed peptide selection. DIA-based retention times and fragmentation patterns provide a 22% and 21% improvement over this, respectively. Using both DIA-based retention times and fragmentation patterns provides a 46% improvement, suggesting that these two factors are additive.

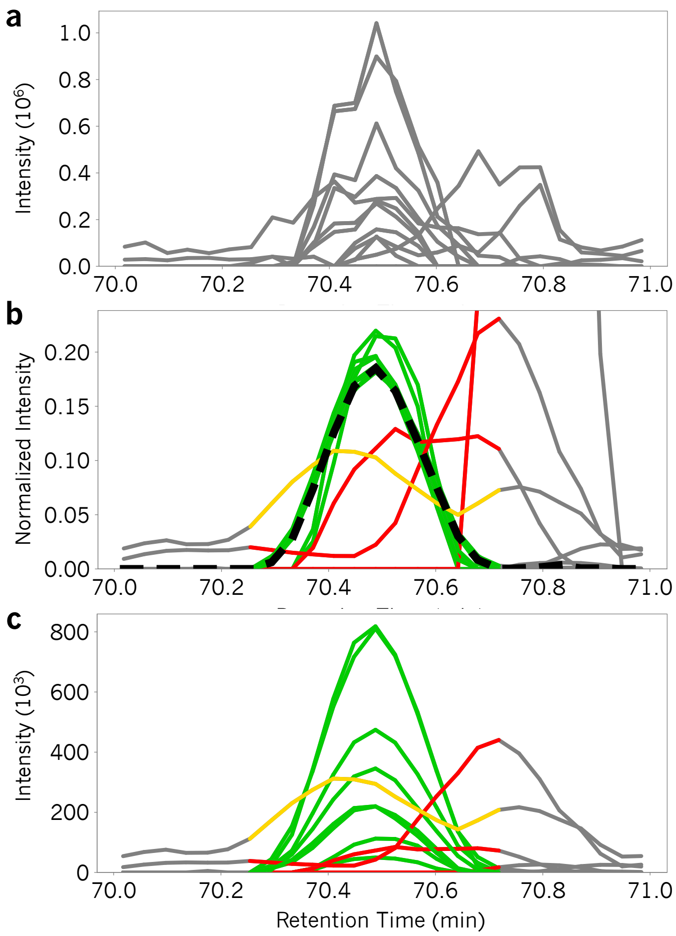

Supplementary Figure 4: Schematic for automated transition refinement. (**a**) After a retention time region for a peptide is detected using the primary EncyclopeDIA score, automated transition refinement is used to determine quantitative fragment ions. (**b**) Briefly, fragment ions are smoothed and normalized such that their area under the curve equals 1. At every retention time point, the median normalized intensity is calculated (dashed line). Normalized intensities that match this shape with a Pearson correlation coefficient >=0.9 are "quantitative" and labeled in green. Normalized intensities that fit with coefficients <0.9 and >=0.75 are labeled in yellow, and those with coefficients <0.75 are in red. (**c**) Quantitative ions can be integrated and summed to approximate the peptide intensity. In general, the median normalized intensity is robust to outliers (transitions with interference) and nonparametrically calculates peak shape without assuming a distribution.

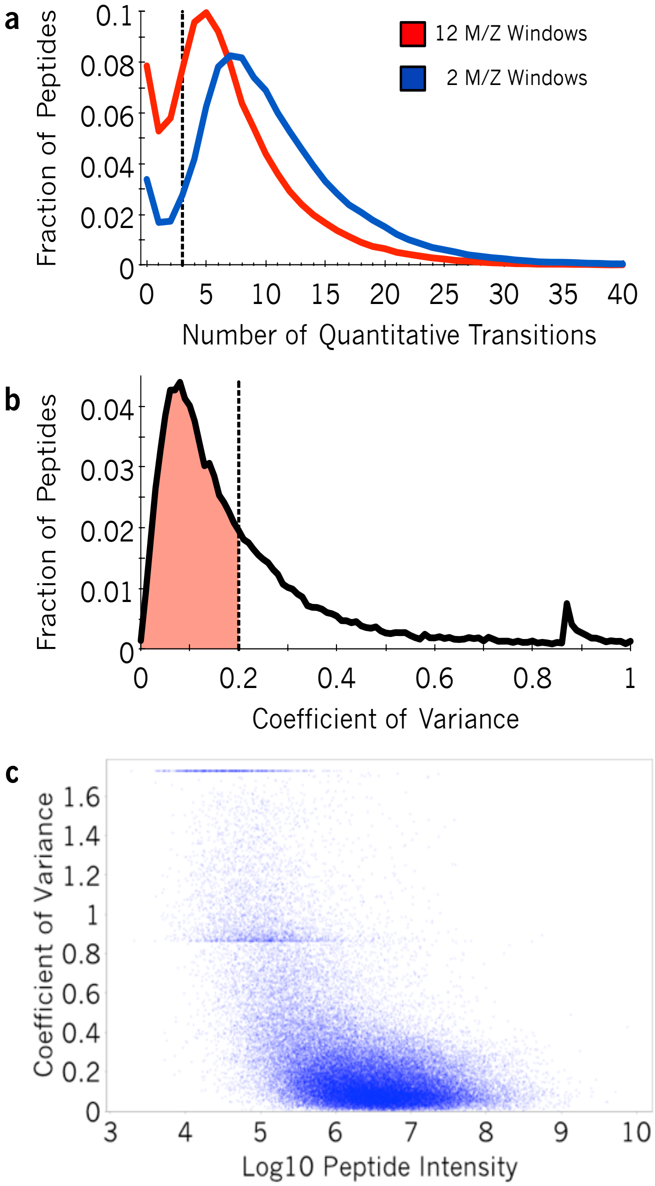

Supplementary Figure 5: Quantitative reproducibility across replicates. (**a**) The number of quantitative transition ions produced by each peptide is greatly affected by precursor isolation window. 93% of peptides detected in narrow-window experiments produce three or more transitions as compared to 81% of peptides in wide-window experiments. (**b**) The distribution of coefficient of variance (CV) in wide-window HeLa replicate experiments: 61% of peptides fit within a 20% CV. (**c**) Coefficient of variance is greatly affected by fragment ion intensity. CV streaks at 0.87 and 1.73 indicate 1 and 2 missing values, which are predominantly from low intensity peptides.

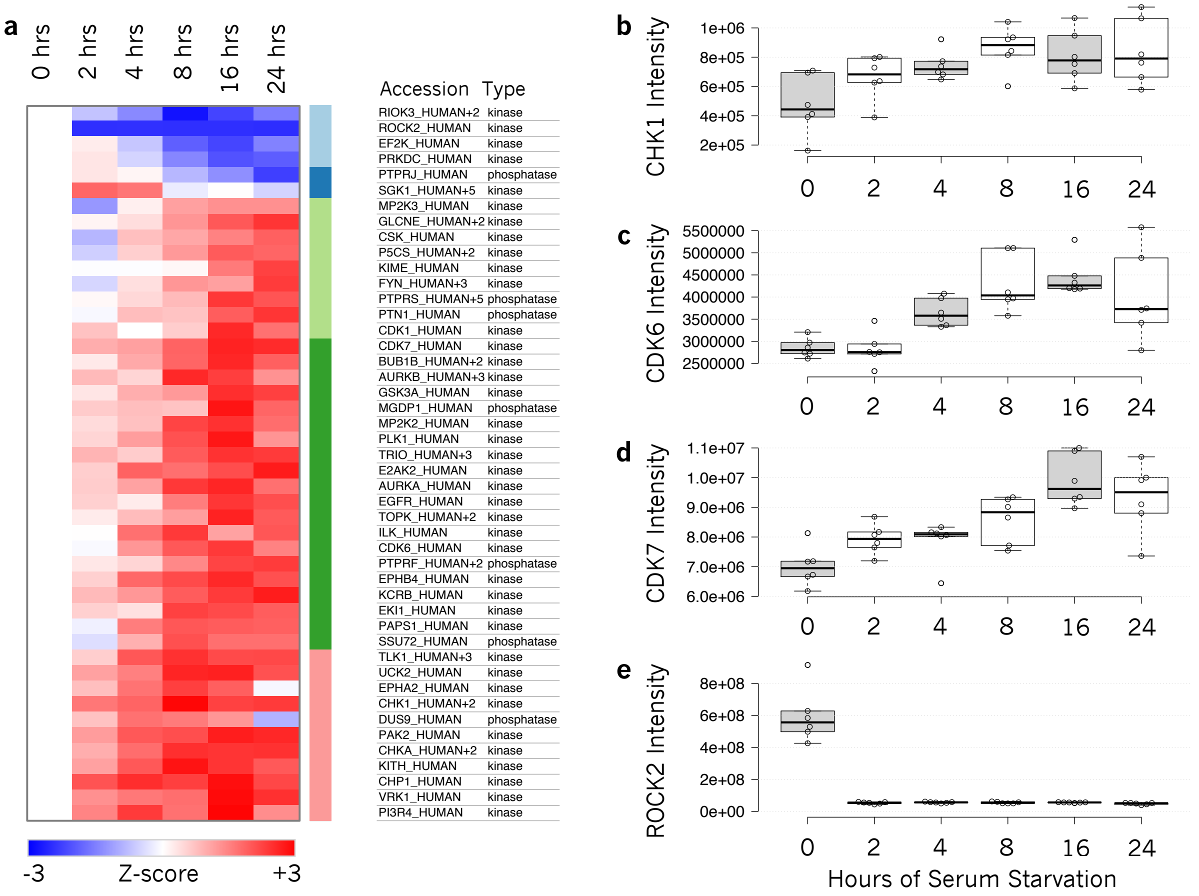

Supplementary Figure 6: Changes in kinase expression following serum starvation. (**a**) Heatmap of 39 kinases and 7 phosphatases found to be quantitatively changing at a q-value<0.01 in HeLa. Colors are Z-score normalized and indicate the number of standard deviations away from the level at time 0. K-means cluster groups are the same as from Figure 4. (**b-e**) Expression changes in known cell cycle regulator kinases (CHK1, CDK6, CDK7, and ROCK2) are out of sync over 24 hour serum starvation.

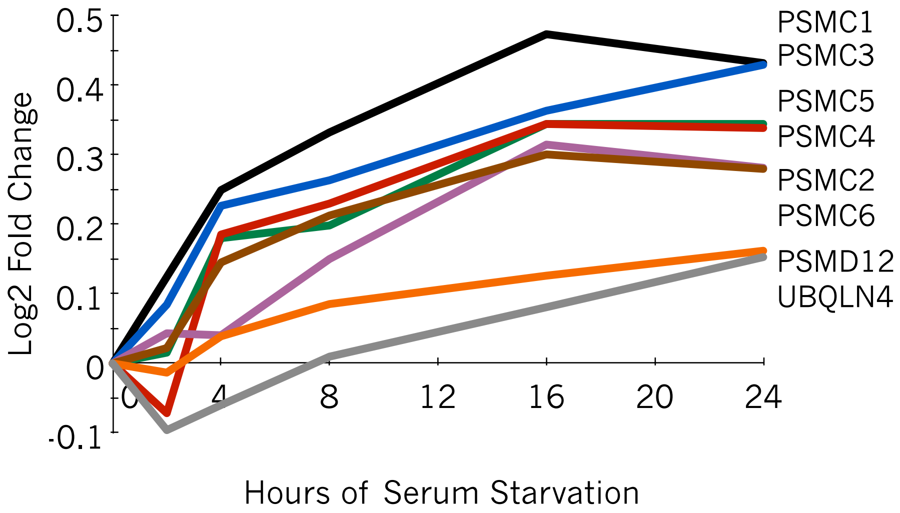

Supplementary Figure 7: Expression changes in proteasome components following serum starvation. All 8 components of the proteasome detected in this study were found to change by approximately 1.25x fold change.

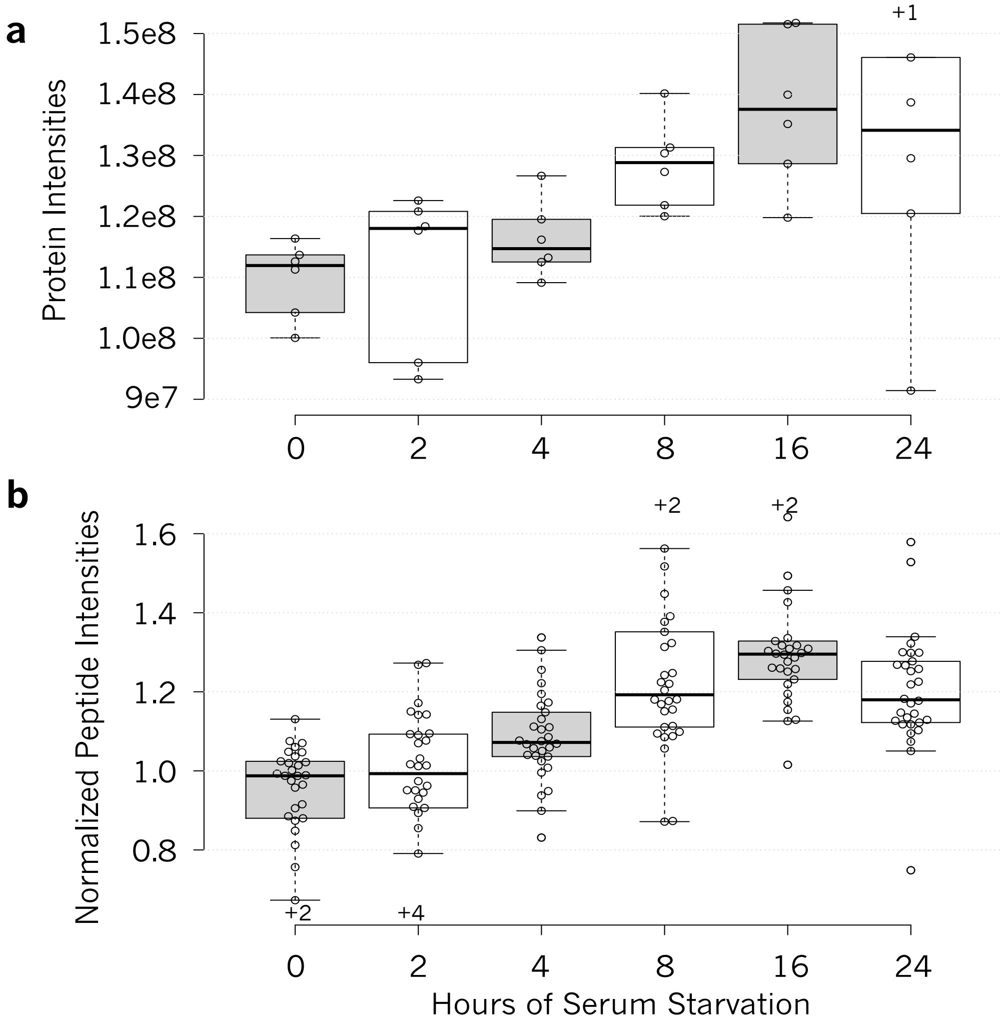

Supplementary Figure 8: Boxplots showing intensity changes in EGFR following serum starvation. (**a**) EGFR protein intensities for 6 replicates over 24 hours of serum starvation indicate an upward trend to a maximum 1.25x fold change at 16 hours. (**b**) Linear model normalized median peptide measurements across 6 replicates for all peptides assigned to EGFR indicate the same trend. Boxes indicate quartiles and bold lines indicate medians. Tukey-styled whiskers extend to data points that are less than 1.5x the interquartile range away from 1st/3rd quartile. All data points are plotted except where indicated on the plot with +X to indicate outliers. Alternating colors simply indicate every other time point.

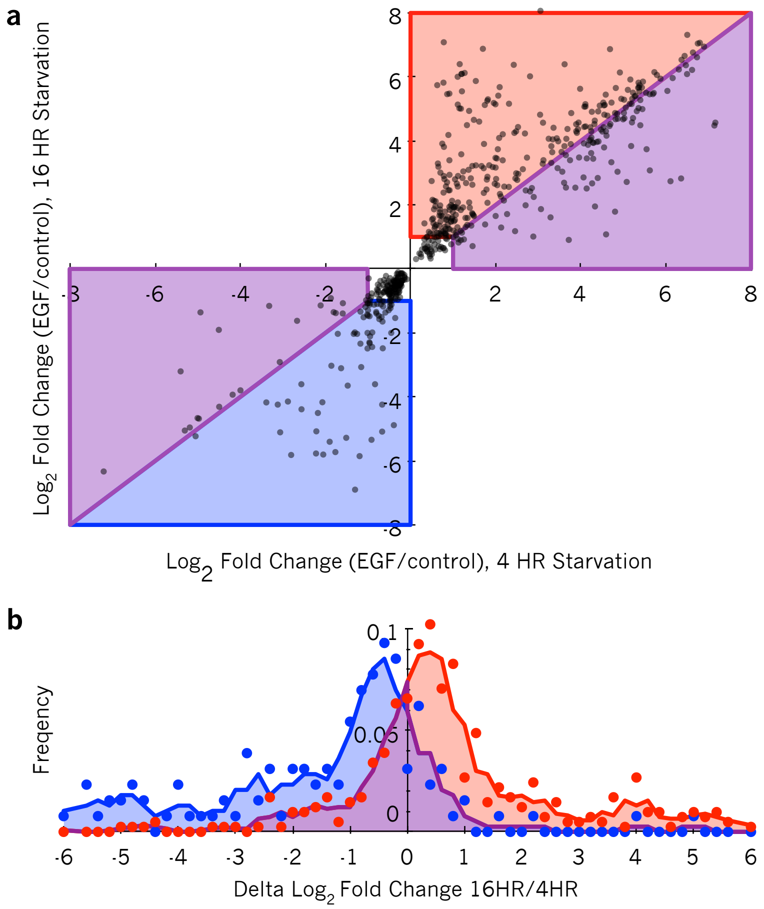

Supplementary Figure 9: Changes in EGF phosphorylation regulation following different serum starvation protocols. (**a**) A scatterplot of phosphopeptides that change significantly as a result of EGF stimulation (FDR<0.05) after 4 hours and 16 hours of serum starvation. If changes in serum starvation were an insignificant perturbation, phosphopeptide response to EGF would fall on a diagonal line. Corroborating our observation of an increase in EGFR, more phosphopeptides fall in the red and blue regions (stronger response after 16 hours starvation) rather than purple regions (stronger response after 4 hours starvation). (**b**) A histogram of delta changes in EGF response from the diagonal line. Dots indicate frequencies binned at 0.2 Delta Log_2 fold change while lines are three-bin moving average smoothed values. The red distribution (matching the red and purple areas in the upper right quadrant) indicates that phosphopeptides that increase after EGF stimulation shows a median of 1.38x increased fold change when comparing a 16 hour starvation versus a 4 hour starvation, correlating with the 1.3x increase in EGFR observed at the proteome level. The blue distribution (matching the blue and purple areas in the lower left quadrant) indicate phosphopeptides that show a median decrease of 0.58x when comparing a 16 hour starvation to a 4 hour starvation and also indicate a sensitization of HeLa to EGF after a longer serum starvation.

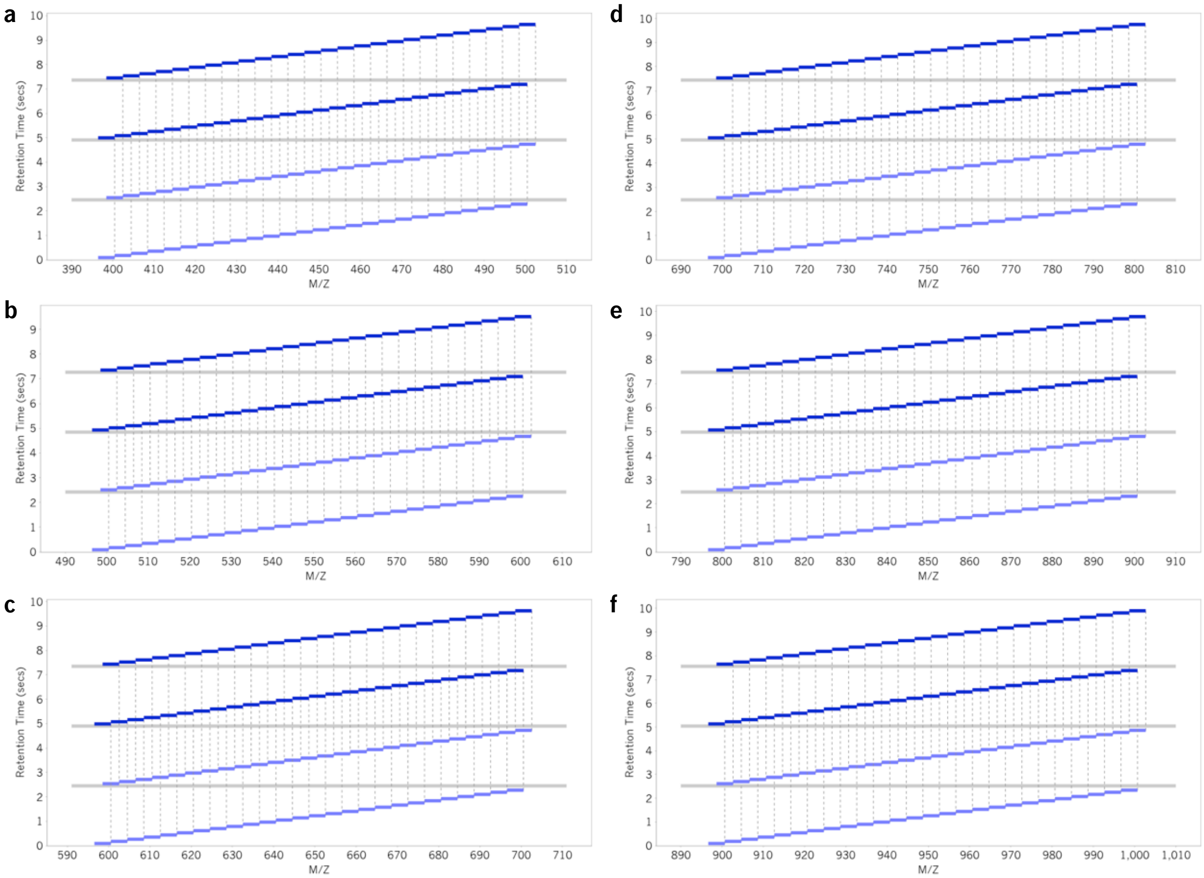

Supplementary Figure 10: Two complete cycles of each narrow DIA precursor isolation windowing scheme. Chromatogram library building DIA experiments use 52x 4 m/z overlapped precursor isolation window cycles to collect fragment ions, with interspersed precursor spectra (gray) every half cycle. The average cycle times were (**a**) 400-500 m/z: 5.00 seconds, (**b**) 500-600 m/z: 4.93 seconds, (**c**) 600-700 m/z: 4.99 seconds, (**d**) 700-800 m/z: 5.05 seconds, (**e**) 800-900 m/z: 5.07 seconds, and (**f**) 900-1000 m/z: 5.13 seconds. In every other cycle for each run the collected windows extend an extra 2 m/z outside of the desired range to ensure that the entire internal range can be completely deconvoluted.

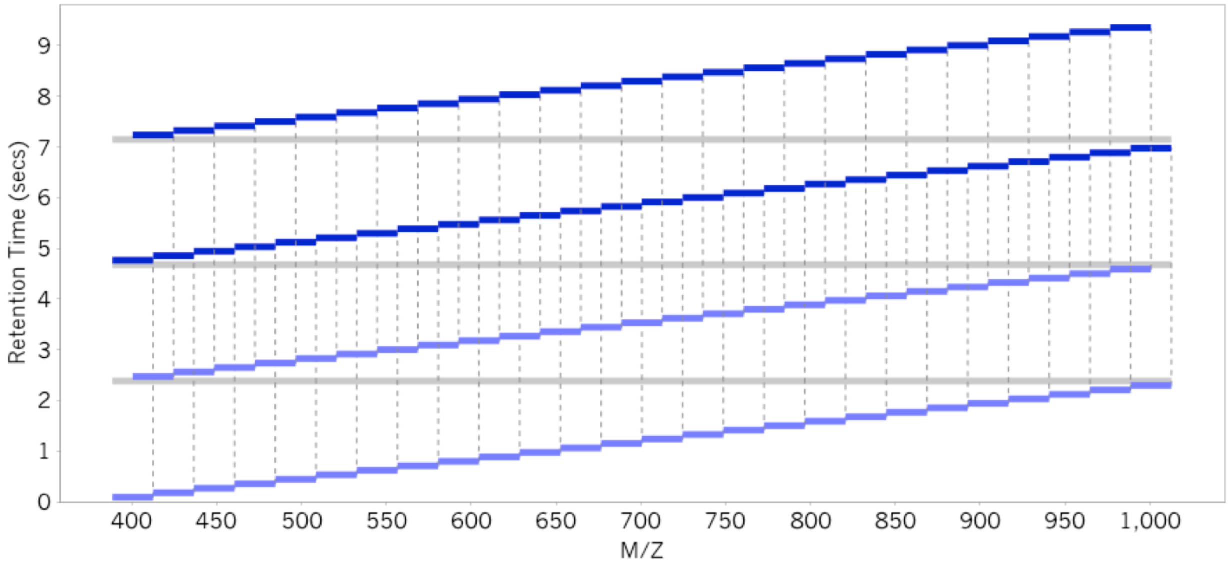

Supplementary Figure 11: Two complete cycles of the wide DIA precursor isolation windowing scheme. Quantitative DIA experiments use 51x 24 m/z overlapped precursor isolation window cycles to collect fragment ions, with interspersed precursor spectra (gray) every half cycle. The average cycle time across all quantitative experiments was 4.74 seconds (min: 4.68 seconds, max: 4.78 seconds). Similar to the narrow window acquisitions, in every other cycle for each run the collected windows extend an extra 12 m/z outside of the desired range to enable complete deconvolution.

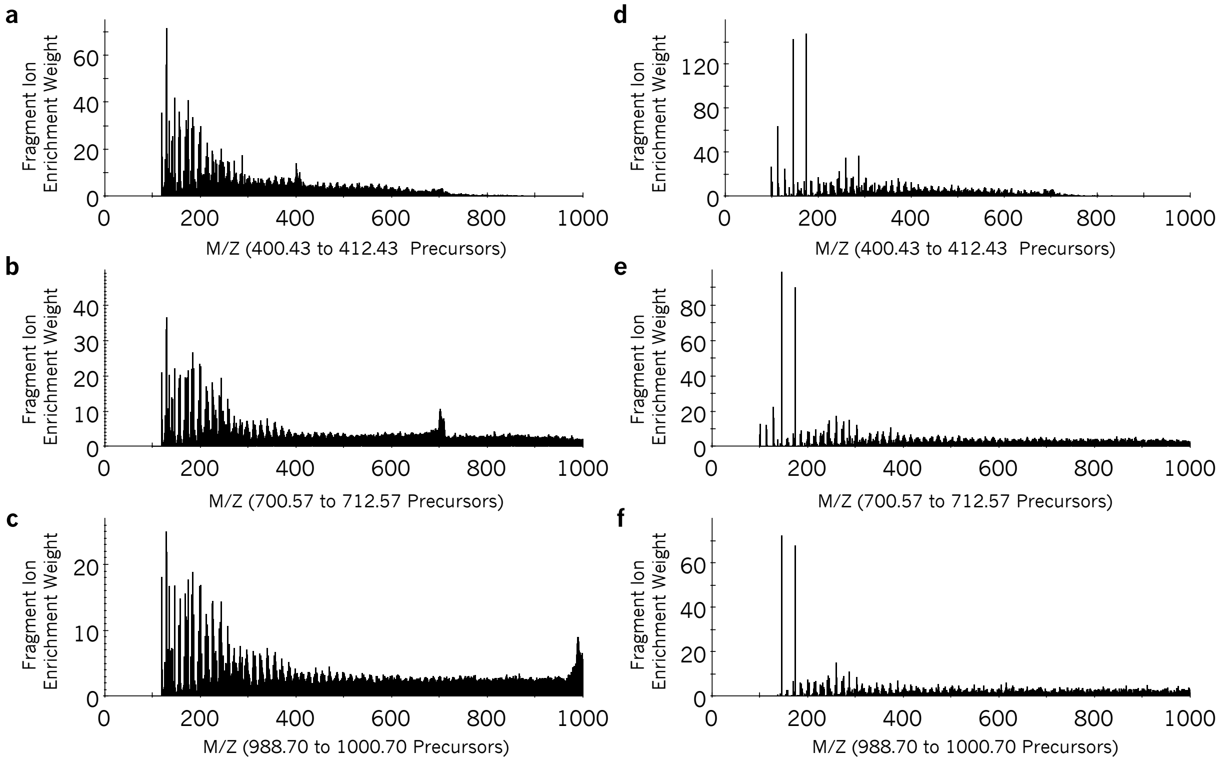

Supplementary Figure 12: Fragment ion enrichment weights. Enrichment weights are normalized frequencies of fragment ion presence/absence (intensity independent) for library peptides in each precursor isolation window. DDA ion frequencies change depending on isolation window, as demonstrated in three different example ranges: (**a**) 400.43 to 412.43 m/z, (**b**) 700.57 to 712.57 m/z, and (**c**) 988.70 to 1000.70 m/z from the HeLa DDA spectrum library. Increased fragment ion frequency in the precursor isolation window is due to unfragmented precursors commonly found in MS/MS experiments. In contrast, DIA chromatogram libraries contain only expected B and Y ions, and consequently are more consistent (**d**) 400.43 to 412.43 m/z, (**e**) 700.57 to 712.57 m/z, and (**f**) 988.70 to 1000.70 m/z. In both cases, ion frequencies increase at lower m/z and are centered around common low m/z B and Y ions.

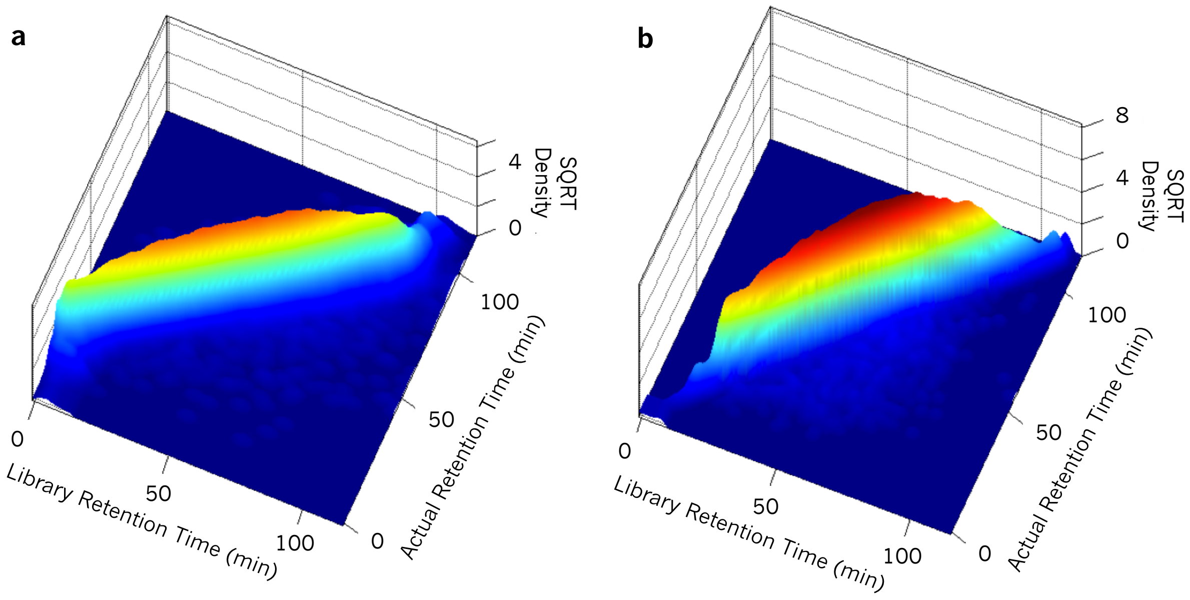

Supplementary Figure 13: **Kernel Density Estimates for Retention Time Alignment.** Three dimensional density plots showing cumulative peptide piles for the DDA spectrum library (**a**) and DIA chromatogram library (**b**) retention time alignments in Figure 4a and 4b. Higher density (Z axis, square root normalized) indicates more peptides at a given retention time point. Treating the density estimate as a mountain range, the retention time alignment approach starts at the point of maximum density and traces the top of the density ridge using a non-parametric ridge walking algorithm to find the optimal alignment. In this approach outliers have relatively low density and generally do not affect the ridge walk algorithm, making it more robust than typical curve fitting strategies.

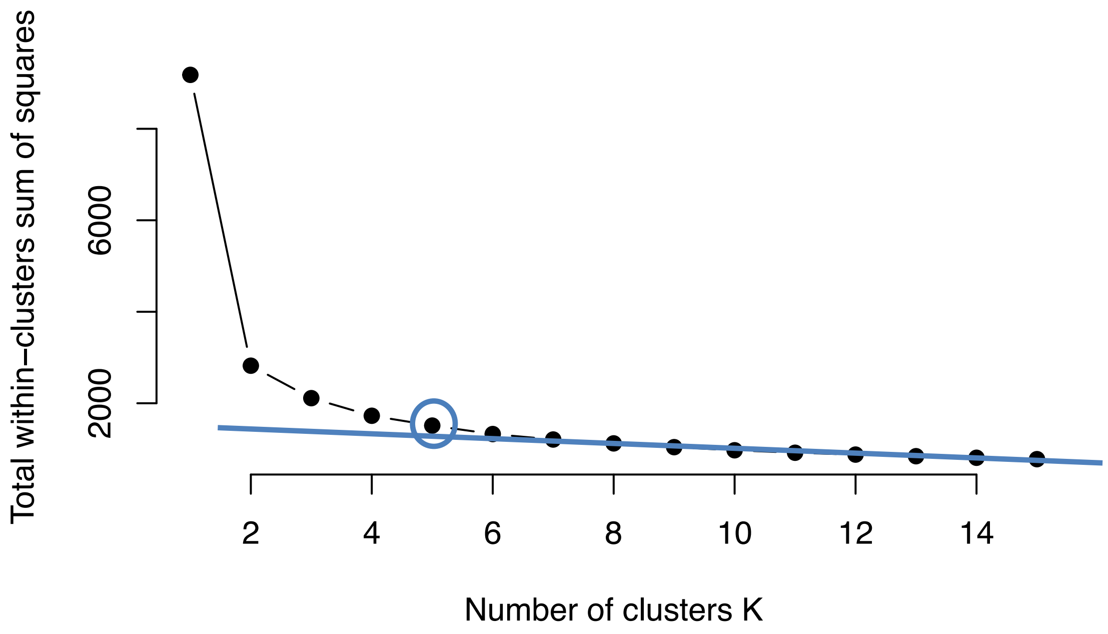

Supplementary Figure 14: Choosing K in K-means clustering. K-means clustering was performed for each K model from 1 to 15 using 1,000 random starting points with 1,000 iterations. We estimated 5 groups by calculating the sum of within squared errors and estimating the first point (highest K) where the change in the sum of within squared errors started to deviate from a flat line.

Supplementary References

1. Ting YS, Egertson JD, Bollinger JG et al. PECAN: library-free peptide detection for data-independent acquisition tandem mass spectrometry data. Nat Methods. 14:903-908 (2017).
2. MacLean B, Tomazela DM, Shulman N et al. Skyline: an open source document editor for creating and analyzing targeted proteomics experiments. Bioinformatics. 26:966-968 (2010).
