# Supplementary material for: Chromatogram libraries improve peptide detection and quantification by data independent acquisition mass spectrometry
Source: Nat Commun. 2018 Dec 3;9:5128. doi: 10.1038/s41467-018-07454-w (PMC6277451; doi:10.1038/s41467-018-07454-w)
Supplement: Supplementary file 7 — Reporting Summary [file 41467_2018_7454_MOESM7_ESM.pdf]

nature research

Corresponding author(s): Michael J. MacCoss

Reporting Summary

Nature Research wishes to improve the reproducibility of the work that we publish. This form provides structure for consistency and transparency in reporting. For further information on Nature Research policies, see Authors & Referees and the Editorial Policy Checklist.

Statistical parameters

When statistical analyses are reported, confirm that the following items are present in the relevant location (e.g. figure legend, table legend, main text, or Methods section).

n/a	Confirmed	
☐	☒	The exact sample size (n) for each experimental group/condition, given as a discrete number and unit of measurement
☐	☒	An indication of whether measurements were taken from distinct samples or whether the same sample was measured repeatedly
☐	☒	The statistical test(s) used AND whether they are one- or two-sided<br>*Only common tests should be described solely by name; describe more complex techniques in the Methods section.*
☐	☒	A description of all covariates tested
☐	☒	A description of any assumptions or corrections, such as tests of normality and adjustment for multiple comparisons
☐	☒	A full description of the statistics including central tendency (e.g. means) or other basic estimates (e.g. regression coefficient) AND variation (e.g. standard deviation) or associated estimates of uncertainty (e.g. confidence intervals)
☐	☒	For null hypothesis testing, the test statistic (e.g. F, t, r) with confidence intervals, effect sizes, degrees of freedom and P value noted<br>*Give P values as exact values whenever suitable.*
☒	☐	For Bayesian analysis, information on the choice of priors and Markov chain Monte Carlo settings
☐	☒	For hierarchical and complex designs, identification of the appropriate level for tests and full reporting of outcomes
☒	☐	Estimates of effect sizes (e.g. Cohen's d, Pearson's r), indicating how they were calculated
☐	☒	Clearly defined error bars<br>*State explicitly what error bars represent (e.g. SD, SE, CI)*

Our web collection on statistics for biologists may be useful.

Software and code

Policy information about availability of computer code

Data collection	Thermo XCalibur 4.0.27.19
Data analysis	Comet version 2015.02v2, ProteoWizard version 3.0.7303, Skyline version 3.1.0.7382, Skyline-daily version 4.1.1.18151, Extraction of Differential Gene Expression (EDGE) 3.6, PANTHER Overrepresentation Test release 20170413, and EncyclopeDIA (https://bitbucket.org/searleb/encyclopedia)

For manuscripts utilizing custom algorithms or software that are central to the research but not yet described in published literature, software must be made available to editors/reviewers upon request. We strongly encourage code deposition in a community repository (e.g. GitHub). See the Nature Research guidelines for submitting code & software for further information.

Data

Policy information about availability of data

All manuscripts must include a data availability statement. This statement should provide the following information, where applicable:

- Accession codes, unique identifiers, or web links for publicly available datasets
- A list of figures that have associated raw data
- A description of any restrictions on data availability

Data availability statement. All mass spectrometry mzML and RAW data files (see Supplementary Data 4 for raw data annotations) are available on the Chorus

Project (project identifier 1433, human [https://chorusproject.org/anonymous/download/experiment/32fa43c0f9ba486eb3eedeb689f87765] and yeast [https://chorusproject.org/anonymous/download/experiment/b98531fe7fe246cbb7e45ce065fe54a9] chromatogram library data, serum starvation data proteomics [https://chorusproject.org/anonymous/download/experiment/e0659292e919414787ec112dca4c57c1] and phosphoproteomics [https://chorusproject.org/anonymous/download/experiment/c24893cd7115446dab4d7eeb7fde2506] data) and at the MassIVE proteomics repository (project identifier MSV000082805 [https://massive.ucsd.edu/ProteoSAFe/dataset.jsp?task=e340c79fbdc64e14a710265761bfeed5]).

Field-specific reporting

Please select the best fit for your research. If you are not sure, read the appropriate sections before making your selection.

- [x] Life sciences
- [ ] Behavioural & social sciences
- [ ] Ecological, evolutionary & environmental sciences

For a reference copy of the document with all sections, see nature.com/authors/policies/ReportingSummary-flat.pdf

Life sciences study design

All studies must disclose on these points even when the disclosure is negative.

Sample size	No sample-size was calculated. Three technical replicates and six biological replicates are typically sufficient for cell line-based studies where biological variability is relatively low.
Data exclusions	No data was excluded from this analysis.
Replication	Global phosphoproteomics after EGF stimulation was used to validate EGFR upregulation as a result of serum starvation.
Randomization	samples were block randomized within each replicate throughout the entire experimental workup.
Blinding	Blinding was not relevant to this study as all samples were block randomized and treated identically after serum starvation or EGF stimulation.

Reporting for specific materials, systems and methods

Materials & experimental systems

n/a	Involved in the study	
[x]	[ ]	Unique biological materials
[x]	[ ]	Antibodies
[ ]	[x]	Eukaryotic cell lines
[x]	[ ]	Palaeontology
[x]	[ ]	Animals and other organisms
[x]	[ ]	Human research participants

Methods

n/a	Involved in the study	
[x]	[ ]	ChIP-seq
[x]	[ ]	Flow cytometry
[x]	[ ]	MRI-based neuroimaging

Eukaryotic cell lines

Policy information about cell lines

Cell line source(s)	HeLa S3 cervical cancer cells (ATCC)
Authentication	Cell lines were authenticated using the STR approach.
Mycoplasma contamination	Cell lines were tested for Mycoplasma.
Commonly misidentified lines (See ICLAC register)	none were used
